# Hourly Wages in Crowdworking: A Meta-Analysis

**DOI:** 10.1007/s12599-022-00769-5

**Published:** 2022-08-30

**Authors:** Lars Hornuf, Daniel Vrankar

**Affiliations:** Faculty of Business Studies and Economics, University of Bremen, Max-von-Laue-Straße 1, 28359 Bremen, Germany

**Keywords:** Crowdworking, Crowdsourcing, Meta-analysis, Hourly wage, Remuneration, Gig-economy

## Abstract

In the past decade, crowdworking on online labor market platforms has become an important source of income for a growing number of people worldwide. This development has led to increasing political and scholarly interest in the wages people can earn on such platforms. This study extends the literature, which is often based on a single platform, region, or category of crowdworking, through a meta-analysis of prevalent hourly wages. After a systematic literature search, the paper considers 22 primary empirical studies, including 105 wages and 76,765 data points from 22 platforms, eight different countries, and 10 years. It is found that, on average, microtasks results in an hourly wage of less than $6. This wage is significantly lower than the mean wage of online freelancers, which is roughly three times higher when not factoring in unpaid work. Hourly wages accounting for unpaid work, such as searching for tasks and communicating with requesters, tend to be significantly lower than wages not considering unpaid work. Legislators and researchers evaluating wages in crowdworking need to be aware of this bias when assessing hourly wages, given that the majority of literature does not account for the effect of unpaid work time on crowdworking wages. To foster the comparability of different research results, the article suggests that scholars consider a wage correction factor to account for unpaid work. Finally, researchers should be aware that remuneration and work processes on crowdworking platforms can systematically affect the data collection method and inclusion of unpaid work.

**Supplementary Information:**

The online version contains supplementary material available at 10.1007/s12599-022-00769-5.

## Introduction

After years of annual double-digit growth rates (Kaganer et al. 2013; ILO 2018), crowdworking has become a multi-billion-dollar industry since its inception in the early 2000s. The attractiveness of crowdworking lies in its business model, which enables the near-instant worldwide matching of workers and requesters on online labor market platforms (Shafiei Gol et al. 2018; De Stefano 2015). Kässi et al. (2021) estimate that 19 million people were active on crowdworking platforms worldwide by the end of 2020, with the number of active workers steadily increasing over the years. A particularly strong increase in crowdworkers recently occurred during the COVID-19 pandemic (Stephany et al. 2020), when many companies closed their offices and working from home became mandatory, especially for the chronically ill. A similar development is also likely to occur from migration due to armed conflicts, which will require access to remote and easily accessible jobs for many ex-employees (Lynn et al. 2021).

At first glance, as an outgrowth of the digital economy, crowdworking provides considerable advantages for workers and requesting companies. Workers are attracted by low entry barriers, high flexibility in working hours and location, and high autonomy in choosing their specific tasks (Hara et al. 2018; Shafiei Gol et al. 2018). These factors can not only promote social mobility, particularly in developing countries and for people with disabilities and other minorities (Kittur et al. 2013; Adams and Berg 2017), but also empower people who have been forced to migrate. Requesters, which are often located in developed countries, profit from remunerations far below the minimum wage in their respective jurisdictions, because workers on internationally operating online labor platforms often come from the Global South, where lower average wages are paid (Agrawal et al. 2015).

However, remuneration in crowdworking has become a source of discontent, due to perceived underpayment on the worker side, regardless of the workers’ location (Whiting et al. 2019). Workers from developed countries are dissatisfied with hourly wages far below the national average. Workers from developing countries can earn wages above the national average in their respective country (Heeks 2017; Berg and Rani 2021) but are often frustrated knowing that their work would be better paid in the requester's country (Berg et al. 2018).

Investigating the hourly wages of crowdworkers is therefore highly relevant, as evidenced by the growing number of studies examining the remuneration on online labor market platforms during the last years. However, research often presents remuneration and work processes in crowdworking only in a simplified and stylized way and rarely differentiates the different categories of crowdwork (Kittur et al. 2013; Jäger et al. 2019). However, the work process often determines the way a worker is compensated and the method of data collection researchers can use. The respective data collection method, in turn, affects whether, for example, unpaid work is taken into account, which can bias the estimated wages. In addition, most studies examining wages on online crowdworking platforms focus on one platform (Beerepoot and Lambregts 2015; Hara et al. 2019) or one region (Dunn 2017; Serfling 2018; Bayudan-Dacuycuy and Kryz Baje 2021) and base their analysis on only one method of data collection (Hara et al. 2018; Wood et al. 2019a). The results of these studies may thus represent non-representative outliers. By conducting a meta-analysis, we overcome many of these limitations and increase the transparency in the hourly wages crowdworkers earn.

We contribute to extant literature in at least three ways. First, we investigate the different remuneration and work processes for various categories of crowdwork. In doing so, we highlight the difficulties and potential biases that various categories of crowdworking might pose to empirical research. Second, we estimate average hourly wages for microtasks and online freelancing using a meta-analysis. Third, we investigate which factors influence the wages of crowdworkers, especially the impact of unpaid labor on hourly wages in crowdworking.

The structure of this article is as follows: Sect. 2 gives an overview of the relevant literature and analyzes the remuneration and work processes for different categories of crowdworking. In Sect. 3, we describe the data and methods, after which we report the results in Sect. 4. In Sect. 5, we discuss the contributions and limitations of our analysis and connect our results with current policy debate. Section 6 concludes the article.

## Literature

### The Crowdworking Wage Debate

Remuneration in crowdworking has attracted increasing public attention, primarily through initiatives by trade unions (Leimeister et al. 2016; DGB 2021), governmental and non-governmental agencies (Dengler and Matthes 2015; FairCrowdWork 2017), and crowdworkers themselves (Salehi et al. 2015; Healy et al. 2020), often supported by research (Deng et al. 2016; Saito et al. 2019; Whiting et al. 2019). Crowdworkers often discuss the conditions on the crowdworking platform and the attractiveness of certain jobs in forums. Crowdworkers also express concerns about this new world of work in academic surveys. Unions and researchers then aggregate these individual voices in best-practice frameworks and catalogs of demands aimed at crowdworking platforms. The platform Fair Crowd Work (www.faircrowd.work), for example, offers trade union information and exchange on crowd, app- and platform-based work. It also offers ratings of working conditions on different online labor platforms based on worker surveys. The Fairwork project (www.fair.work) of the Oxford Internet Institute, University of Oxford, and the Berlin Social Science Centre also evaluates the working conditions of online labor platforms worldwide. In this way, unions in particular help overcome the power asymmetry between workers and platforms, which would otherwise leave an individual crowdworker with minimal bargaining power (Kingsley et al. 2015; Auer et al. 2021). A review of guidelines, codes of conduct, and standards proposed by researchers, unions, and workers shows that poor remuneration is the most frequently faced problem of crowdworkers, often linked to demands for a minimum wage (Heeks et al. 2021).

The response of crowdworking platforms to these demands varies, with some platforms not even willing to discuss them on a regular basis (Gegenhuber et al. 2021). Most platforms disclaim responsibility for the remuneration of workers completing tasks on their platforms, as they define themselves as mere intermediaries between requesters and workers (Cunningham-Parmeter 2019; Wei and MacDonald 2021; Tay and Large 2022). The remuneration of crowdworkers is then entirely at the discretion of the requester, with the exception of some platforms that implemented a minimum hourly wage, such as Upwork ($3) (Heeks 2017). Some companies have also signed self-commitments to improve the working conditions on their platforms (Funke and Picot 2021), voluntarily offer workers the opportunity to gain further qualifications through tutorials, and actively encourage the exchange between workers within the framework of best practices (Mrass et al. 2018).

While legal scholars and legislators have focused on aspects such as the applicability of labor law and social security law to crowdworkers (De Groen and Maselli 2016; Greef et al. 2017; Schoukens 2020; European Commission 2021), social scientists in particular have raised awareness of wage levels in crowdworking. Initial research has mostly focused on the requester side of online labor market platforms and, for example, determined the reservation wage of workers completing microtasks (Horton and Chilton 2010). Moreover, platforms such as Amazon Mechanical Turk (MTurk) not only are the subject of investigation but also have become increasingly popular as a low-cost way to obtain samples for empirical research projects. As wage transparency has increased, with many empirical articles informing workers and legislators about the wage levels of crowdworkers, concerns have grown about underpayment and exploitation of workers by both the private sector (Paolacci et al. 2010; Nickerson 2013; Pallais 2014; Brawley and Pury 2016) and the academic sector (Silberman et al. 2018; Shmueli et al. 2021).

### Remuneration of Crowdworkers and Work Processes

To understand how crowdworkers and requesters set wages, we outline the different remuneration and work processes in online crowdworking in Table 1, which gives an overview of different crowdworking categories and exemplary tasks and platforms. We base the categories and definitions on the work of De Stefano (2015), Kuek et al. (2015), and Boudreau and Lakhani (2013). Crowdworking research often collects data on hourly wages using one of two methods: *surveys* (e.g., Wood et al. 2019a; Giard et al. 2021) and *technical data collection methods* (e.g., Ipeirotis 2010; Hara et al. 2018). The overview of different crowdworking categories shows how different work processes in microtasks, online freelancing, and crowd contests influence the choice of data collection methods.^1^ The more complex the work processes, especially if they require a high level of offline work and thinking, the more difficult it becomes to collect wages with technical methods. Researchers then must resort to surveys or rely on the cooperation of platforms to share primary data.

**Table 1:** Categories of online crowdwork

Category	Description	Exemplary tasks	Exemplary platforms
Microtasks	Microtasks consist of small, repetitive tasks that require minimal cognitive effort and little to no interaction with requesters. Remuneration is earned per task.	Data entry, digitizing, image recognition, surveys, web research	Clickworker, Crowdflower, Microworkers, MTurk, Prolific, Taskrabbit, Toloka
Online Freelancing	Online freelancing tasks often require a distinct skill set from workers. Usually, they require communication with requesters. Remuneration is often paid per hour but can also be earned per task.	Programming, translating, legal advice, administration	Fiver, Freelanced, Freelancer, oDesk, PeoplePerHour, Upwork
Crowd Contests	Crowd contest are competitions in which participants submit their work. Interaction with requester is minimal. Remuneration depends on the client that ranks the work submitted. Therefore, a worker may not receive remuneration, despite completing the work.	Designing a logo or a web page, solving a company’s problem	110 designs, 99 designs, DesignCrowd, GoPillar, Hatchwise, HYVE, Topcoder
Crowd Complementor	Crowd complementors offer products, software, or services within an ecosystem built and maintained by a company and thus generate value for the company, as well as for users in that specific ecosystem. The remuneration is usually subject to a fee charged by the company providing the ecosystem or platform.	Developing an app, recording a video, uploading a song or photo	Google Play Store, iTunes, Soundcloud, YouTube
Collaborative Community	Collaborative communities are often dedicated to a greater purpose. Activities are often unpaid and performed as a hobby, which instead of money pays off in terms of recognition in the respective community.	Developing open-source software, translating, helping other users on the same platform	Apache, Translate, Facebook, Wikipedia

The crowdworking categories are based on De Stefano (2015), Kuek et al. (2015), and Boudreau and Lakhani (2013). For an overview of the size of individual platforms, see Kässi and Lehdonvirta (2018)

Microtask completion involves the simplest remuneration and work process, which entails pay per task. Work classified as microtasks often involves assignments that take only seconds or a few minutes to complete and require only little prior knowledge and rudimentary education (Gao et al. 2015; Schmidt 2017; Durward et al. 2020). Tasks range from data entry and transcription to image recognition. The most notable platforms are MTurk and Appen (Rani et al. 2021). It is precisely this category of crowdwork that some scholars view as an extreme form of Taylorism (Kittur et al. 2013; Aloisi 2015), defined as the partitioning of a large, intellectually demanding task into many small tasks, each of which can be completed with minimal mental effort. Although the work process for microtasks clearly contributes to the dehumanization of workers (Kittur et al. 2013), the extremely short completion cycles per task and low skill requirements create flexibility, enabling workers to fill otherwise unproductive times of their day (Chandler and Shapiro 2016).

**Fig. 1:**
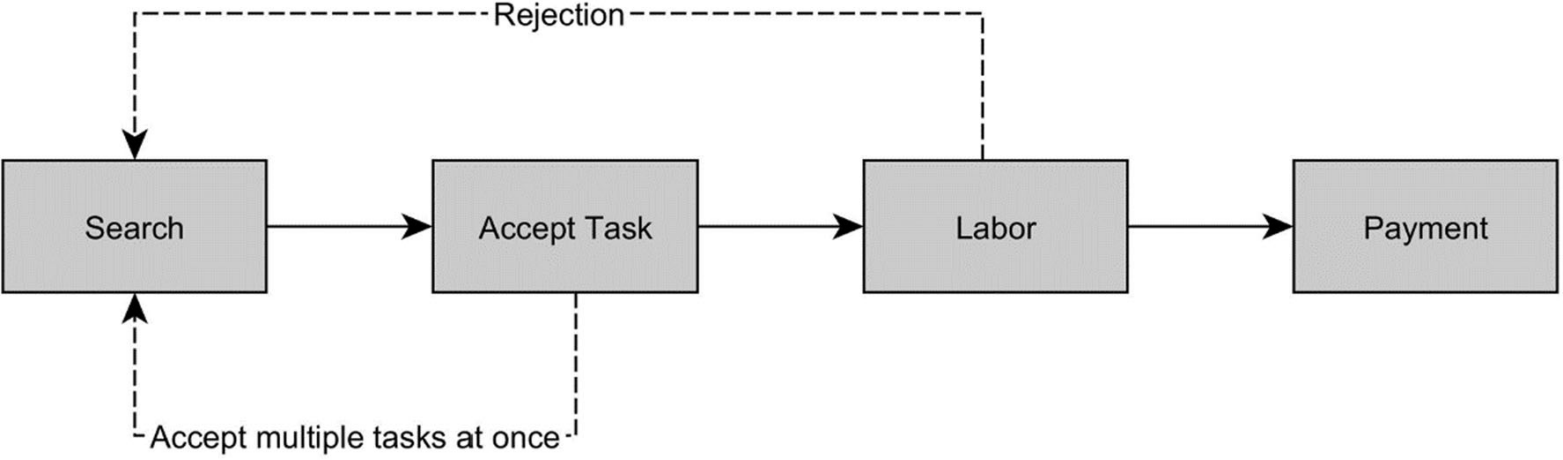
Work process for microtasks. Dotted lines show possible divergences from the standard work process and might be platform specific

The work process for microtasks is extremely standardized and designed to minimize direct communication between employees and customers, as Fig. 1 shows. Workers are rarely invited by requesters to participate in certain tasks (Berg 2016), instead mainly searching themselves for tasks posted by requesters. After workers encounter a task they want to complete, they can accept the task and either begin work directly or open additional tasks, thereby preventing the already-opened task from being assigned to another worker (Hara et al. 2018). A common practice is to take on multiple jobs at once, as this allows workers to reserve well-paying work. Nevertheless, crowdworkers must complete each of the accepted tasks within a deadline set by the requester; otherwise, the task will be made available again to all workers (Toxtli et al. 2021). When a task is completed, workers wait for the requester to accept their work to receive the promised payment. However, on most platforms, requesters can reject workers' submitted tasks with minimal or no feedback (Lascău et al. 2022), though requesters are allowed to keep the results of the rejected work (Beerepoot and Lambregts 2015). Requesters benefit because, due to the low remuneration per task, it is hardly worthwhile for crowdworkers to invest their time in efforts to contact the requesters to find out why their work was rejected (Berg et al. 2018).

Although the remuneration and work process related to microtasks allow for some variation as a result of rejections and the simultaneous assignment of multiple tasks, they are usually very similar and best suited for large-scale, standardized, technical data collection methods. On MTurk in particular, researchers have created plugins and use them to collect wage data. After a worker has installed one of the available plugins, the plugin tracks the completion time per task, the reward per task, and the acceptance rate, among other statistics, and allows the estimation of an hourly wage (Callison-Burch 2014; Hara et al. 2018). The early plugins simply divided the remuneration per task by the duration of the task to estimate an hourly wage. While unpaid work was generally neglected, newer plugins also include components of unpaid work in their calculations and thus can provide a more accurate picture of hourly wages (Hara et al. 2018; Toxtli et al. 2021).

While researchers collect data from the plugins they develop, various features encourage workers to install them for free. First, the calculated hourly wage is displayed to workers, helping them keep track of their productivity.^2^ Second, some plugins offer crowdworkers the ability to rate each requester after completing a task. These ratings are then aggregated and made available to other crowdworkers using the same plugin. In this way, workers can potentially be warned about tasks that the community considers unfair or unfeasible, reducing information asymmetry on crowdworking platforms (Irani and Silberman 2013; Agrawal et al. 2015; Saito et al. 2019). Overall, technically collected data on hourly wages related to microtask platforms have the advantage of reflecting the remuneration actually paid to crowdworkers; by contrast, surveys can be subject to biases in human memory or perception (Moore et al. 2000; Choi and Pak 2005).

In the second category of crowdworking, online freelancing, technical data collection methods are used less frequently when examining wages, which are determined by a somewhat more complex remuneration and work process. Online freelancing entails assignments that can take hours, days, and even weeks and need specialized skills, such as programming knowledge, the comprehension of multiple languages, or legal expertise (Beerepoot and Lambregts 2015). Exemplary tasks involve designing a logo, developing a small computer program, or acting as customer support for a requester’s product. As a result, a disproportionate number of workers on online freelancing platforms have earned at least a bachelor’s degree (Ross et al. 2010; Bertschek et al. 2016; Rani et al. 2021). The mismatch between the average education of the general population and the people who work as online freelancers is especially high in developing countries (Berg et al. 2018; Braesemann et al. 2021). Because of the specific skill requirements in online freelancing, building longer-term relationships with workers who have done a good job in the past is often advantageous for requesters. Longer-term relationships between requesters and workers are therefore more common in online freelancing than for microtasks (Rani and Furrer 2019; Idowu and Elbanna 2021).

**Fig. 2:**
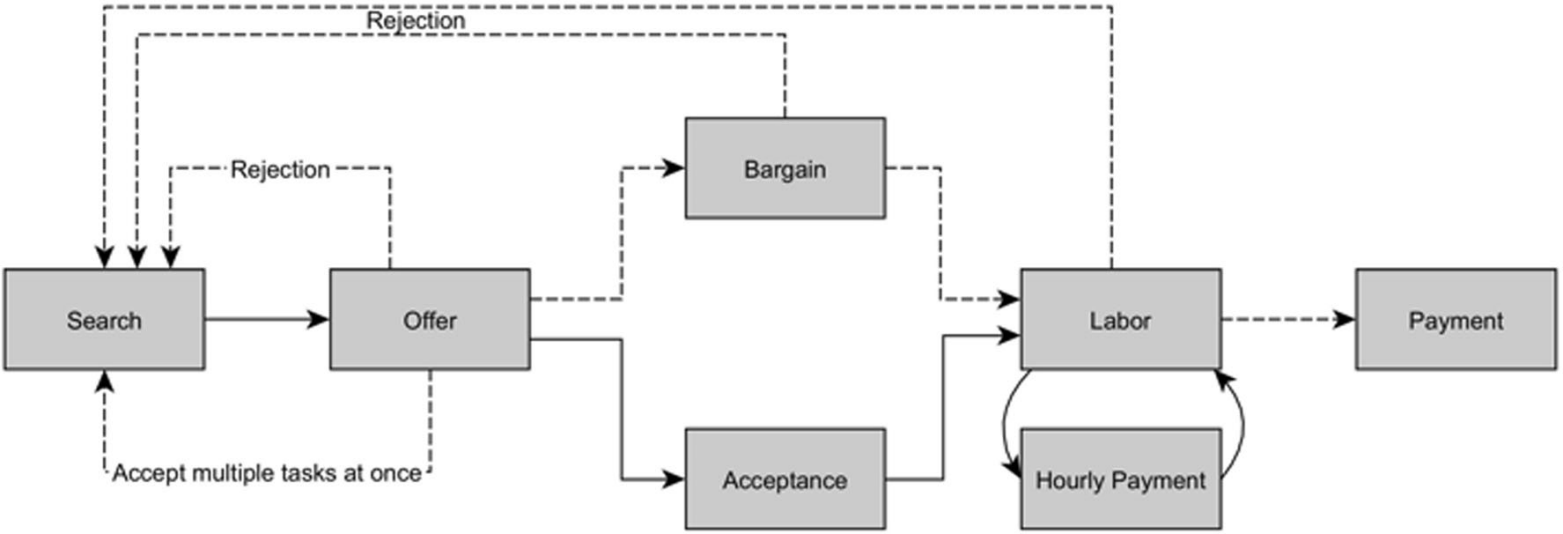
Work process in online freelancing. Dotted lines show possible divergences from the standard work process and might be platform specific

The work process often begins with a freelancer searching for work or with an invitation from a requester (see Fig. 2). Both cases lead to an offer from the online freelancer for an hourly wage or a proposal for a total remuneration (Prassl and Risak 2016). The requester can directly reject or accept the offer, but usually the online freelancer and the requester further negotiate the remuneration (Beerepoot and Lambregts 2015; Fabo et al. 2017). In the case of online freelancing, the requester can also reject the task after the work has been completed. However, online freelancing platforms have a more detailed dispute resolution system than microtask platforms (Jarrahi et al. 2020; Lee and Cui 2020).

Because wages on online freelancing platforms are not publicly negotiated and remuneration and work processes are not as standardized as microtasks, researchers must either examine wages through surveys with crowdworkers or source the negotiated wages from the platforms themselves. This situation makes researchers dependent on the willingness of crowdworkers to participate in surveys or online labor platforms to participate in research projects and to share primary data (Agrawal et al. 2015; Barzilay and Ben-David 2017; Dunn 2017). Moreover, plugins that collect wage data from online freelancers could provide skewed wage estimates, as a greater proportion of unpaid work goes undetected. Web-scraped data that investigate the wages requested by the workers on their profile potentially deliver biased estimates of realized wages, because paid wages can be approximately 25% lower than the remuneration initially asked for by the worker after bargaining (Beerepoot and Lambregts 2015). Moreover, another reason why web-scraped data potentially deliver biased estimates is that workers requesting lower wages might complete more tasks than workers that request higher wages. It would therefore be a mistake to give equal weight to each wage requested on a platform.

**Fig. 3:**
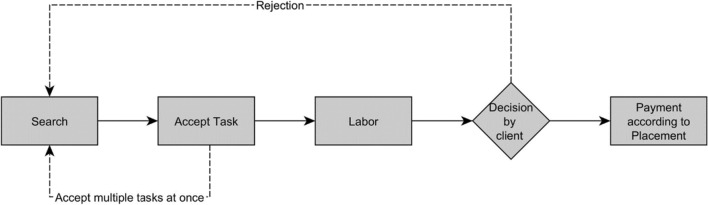
Work process in crowd contests. Dotted lines show possible divergences from the standard work process and might be platform specific

Obtaining wage data is even more difficult in crowd contests, the third category of crowdworking. Up till now, surveys have been the only way to determine the wages of workers participating in crowd contests (De Groen and Maselli 2016; Leimeister et al. 2016). While the work process is easily explained and visualized in Fig. 3, determining an hourly wage is more difficult. In crowd contests, workers search for tasks on platforms such as 99 designs or HYVE and submit one or multiple solutions to the contest. The complex tasks range from designing a logo to solving a certain technical problem, which can require specific skills in areas such as medicine, chemistry, or engineering (Boudreau and Lakhani 2016). Payment is based on a rank placement entirely determined by the requester (Segev 2020). Depending on the platform, only the worker with the best solution receives a remuneration or the amount is distributed to the first places in descending order (Rani et al. 2021). In this type of work, determining when the crowdworker is actually working is particularly difficult. For example, while the time required to draw a logo can be measured, it is questionable whether a reliable working time can be determined for complex problems that require a high degree of mental work, such as developing a better algorithm for film suggestions on Netflix.

Finally, crowd complementors, such as app developers, often receive a fixed fee for their contribution that has been *ex ante* defined by the platform. Collaborative community platforms often involve innovation contests among regular employees of a company or users of a product who receive no additional compensation for their activities on the platform (Boudreau and Lakhani 2013); as such, we do not explicitly discuss these processes here.

## Data and Method

### Data

**Fig. 4:**
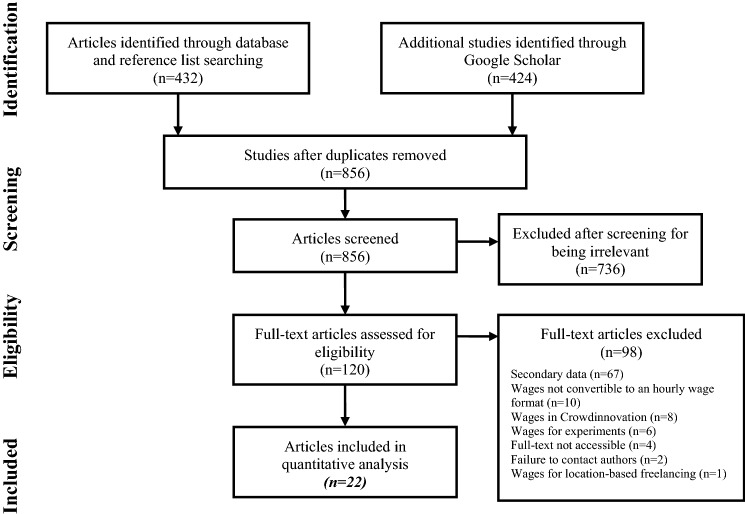
PRISMA flow diagram. This flow diagram reports how the 22 primary studies in the meta-analysis were selected for inclusion. It is based on the template of Liberati et al. (2009)

Our empirical analysis focuses on online crowdwork; we do not examine location-based platforms such as Grubhub, TaskRabbit, or Uber. To investigate wages in crowdworking, we conduct a meta-analysis in line with the guidelines for Preferred Reporting Items for Systematic Reviews and Meta-Analyses (PRISMA) and use a slightly modified template from Liberati et al. (2009). We provide a flow diagram in Fig. 4 of the process of searching, screening, and including or excluding studies in the empirical analysis. As a first step in our meta-analysis, we conducted a systematic literature search from July 2020 to March 2021, which we subsequently updated in March 2022, to identify suitable studies and hourly wages. Initial keywords were extracted from *ex ante* known articles that analyze hourly wages in crowdworking. With keyword combinations such as “crowdwork per hour,” “crowdsource remuneration,” “crowdwork earnings,” and “crowdwork hourly,” (cf. Online Appendix A, available online via http://link.springer.com) we then manually searched the databases ScienceDirect, Scopus, Business Source Premier, and ProQuest,^3^ which led to the identification of 432 potentially relevant studies and articles. In a second step, we considered the first 50 search results on Google Scholar for all the keywords and keyword combinations, which resulted in 424 additional studies found during the systematic literature search. We judged studies as potentially relevant if they were published in a journal, as a report of a trade union, by a government authority or non-governmental organization, or as a conference paper. Of the 856 potentially relevant studies, 736 were irrelevant, because they did not report a crowdworking wage in any form. Consequently, we checked the remaining 120 studies for eligibility and searched their respective reference lists, to minimize the risk of missing an important observation.

To be included in the meta-analysis, publications needed to meet two criteria. First, a relevant study had to state an hourly wage or allow the calculation of an hourly wage on the basis of primary data collected by the respective authors of the study. If, for example, the average weekly wage and the average weekly work hours were reported, we calculated the hourly wage and included the study in the meta-analysis. Second, a study must have specified the number of data points on which the reported wage was based. For 67 studies, the reported wages were collected by other researchers and not by the respective authors. Eight studies stated prizes for crowd contests without giving any information on the work time spent for winning the contest.

One study mixed survey responses from online freelancers with responses from freelancers working on *location-based* platforms (Rani and Furrer 2021) such as Uber or Lieferando and therefore was excluded. Six studies stated hourly wages the authors paid in experiments on online labor market platforms. These studies did not have the goal to collect primary data on crowdworking wages, and therefore we did not include them in the meta-analysis. We contacted the authors of five studies to obtain additional statistics and, in the end, considered three of the studies. We were unable to obtain full text versions of four studies. Finally, we calculated 10 hourly wages from other statistics in the respective article. Seven observations initially measured in euros were converted into U.S. dollars, with the exchange rate at the time of data collection of the respective study.

Overall, 22 primary studies were eligible for inclusion in the meta-analysis, in which we made 105 *observations* of hourly wages*.* Observations are the average hourly wages reported in a given study for a sample of crowdworkers. The 105 average hourly wages that represent the observations are based on 76,765 *data points*. Each data point represents either the response to a survey question about the wage of a crowdworker or a wage collected via a technical data collection method on the respective platform. Unless it was explicitly stated that unpaid work was not considered, we decided to count observations from surveys as considering unpaid work. We also decided to include multiple observations per study in our dataset if, for example, a study included paid and unpaid wages of the same workers. We used this approach to prevent the loss of additional information (Bijmolt and Pieters 2001); however, we did not conduct any empirical tests with overlapping information from the same study. The sample includes between one and 22 hourly wages per study, while the mean was five hourly wages per study. The average number of data points per study was 3489 and varied between 14 and 12,326 data points. We account for the variance in data points per study in a weighting procedure, which we describe in more detail in Sect. 3.3. Table 2 provides an overview of the included studies and the regions and platforms they respectively cover.

**Table 2:** Studies included in the empirical analysis

Study	Country/region	Platform	Wages^h^	No. of data points	
				Paid	Unpaid
Bayudan-Dacuycuy and Kryz Baje (2021)	Philippines	–	$4.60	–	381
Beerepoot and Lambregts (2015)	U.S	oDesk^a^	$3.11–$26.66	925	–
Berg (2016)	U.S., India, International	MTurk, CrowdFlower,^b^ Prolific, Microworkers	$1.90–$7.60	1056	1056
Berg et al. (2018)	U.S., India, International	MTurk, CrowdFlower,^b^ Prolific, Microworkers	$2–$8.50	2020	2022^c^
De Groen et al. (2016)	Italy, Serbia	CoContest^d^	$3.50–$10.30	156	–
Dunn (2017)	U.S	“One of the largest online platforms for work”	$10.64–$15.29	12,932	–
Giard et al. (2021)	Germany	“Marketplace, Microtask”	$6.16–$9.54	–	379
Hara et al. (2018)	International	MTurk	$3.13–$3.48	2666	5332
Hara et al. (2019)	U.S., India	MTurk	$2.48–$3.47	1113	–^e^
Ipeirotis (2010)	International	MTurk	$4.80	5147	–
Jiang et al. (2021)	International	MTurk	$5.12	–	260
Kaplan et al. (2018)	U.S	MTurk	$4.73–$5.12	–	720
Leimeister et al. (2016)	Germany	“Microtask, marketplace, design, testing”	$5.94–$15.45	–	248
Litman et al. (2020)	International	MTurk	$4.59–$4.87	22,272	–
Pallais 2014	International	oDesk^a^	$2.11–$2.20	3767	–
Rani and Furrer (2021)	Africa, Asia, Latin America,	MTurk, CrowdFlower,^b^ Clickworker, Prolific, Microworkers	$1.30–$5.80	1350	1350 ^c^
Rani et al. (2021)	China, Ukraine, International	^f^	$2.70–$11.20	1983	1988
Barzilay and Ben-David (2017)	U.S	Upwork	$17.26–$58.96	4324	–
Ross et al. (2010)	International	MTurk	$1.67–$1.92	–	1823^g^
Saito et al. (2019)	International	MTurk	$9.15	83	–
Wong et al. (2020)	International	MTurk, Clickworker	$5.56	801	–
Wood et al. (2019a)	Africa, Asia	"On one of two leading platforms"	$3.66–$4.41	–	611
				∑ 60,595	∑ 16,170

^a^The two crowdworking platforms Elance and oDesk merged in 2013 and resulted in a new platform called Upwork

^b^CrowdFlower was acquired by Appen in 2019

^c^Dataset is from the ILO 2017 survey

^d^CoContest Inc.'s contest website is now called GoPillar

^e^Dataset is from Hara et al. (2018)

^f^Freelancer, Upwork, 99designs, 680, EPWK, k68, ZBJ, Advego.ru, MTurk, fl.ru, Free-lance.ua, Freelance.ru, Freelance.ua, Freelancehunt.com, Freelancer.com, Kabanchik.ua, Upwork.com, Weblancer.net, and Other

^g^Authors state that their data are out of date and should no longer be used

^h^Column contains the raw data extracted from each source. The raw data can be found in the respective source and is not adjusted for inflation

Our meta-analysis includes at a minimum 15,580 unique workers.^4^ This figure is the result of adding up data points from different studies but considering only the study with the most underlying data points in the respective country, which hardly includes the same respondents. Two studies in our sample use the same dataset from a 2017 International Labour Organization survey (Berg et al. 2018; Rani and Furrer 2019). Moreover, Hara et al. (2018) and Hara et al. (2019) use identical primary data sources for their studies. We included all these studies in our meta-analysis because they offer different insights into the same datasets. For example, Berg et al. (2018) make a distinction between the mean wage of American and Indian workers on MTurk, and Rani and Furrer (2019) provide the mean wage for the entire Asian region. As a general rule, when calculating mean hourly wages in our meta-analysis, we made sure to only include observations based on different primary datasets. If we confronted multiple observations from the same dataset (e.g., Hara et al. 2018, 2019), we used the observation with the most underlying data points to account for overrepresentation bias (Revelli and Viviani 2015).

In total, we obtained hourly wages for workers from eight different countries,^5^ working on 22 of the most common online labor market platforms^6^ (Kässi and Lehdonvirta 2018), and 10 years.^7^ Scholars obtained roughly three-quarters of the observations through technical data collection methods, and approximately one-fifth of all observations account for unpaid work. Therefore, to the best of our knowledge, our meta-analysis uses the most comprehensive dataset of hourly wages in crowdworking in the literature.

### Variables

Table 3 provides an overview of the variables we use in our empirical study. The variable *Hourly Wage 2021* is the variable of interest and measures the hourly wage of crowdworkers adjusted for the year 2021 in U.S. dollars. We adjust hourly wages to allow for meaningful comparisons between wages measured at different points in time, using the inflation rate of the respective country where the data was collected. If no inflation statistics for the specific country or region were available, we considered the international inflation rate (International Monetary Fund 2021).

**Table 3:** Definition of variables

Variable	Definition
Data collection method	Dummy variable that equals 1 if the data points of the respective observation were obtained through technical data collection methods (e.g., a browser plugin) and 0 if the authors conducted a survey.
Crowdworking category	This categorical variable indicates the category of crowdwork from which the wage was collected. It is equal to 1 if the observation results from workers completing microtasks, 2 if it results from online freelancers, and 3 if it was collected for a worker participating in a crowd contest.
Involves unpaid work	Dummy variable that equals 1 if the respective observation considers unpaid work and 0 otherwise.
Hourly wage 2021	The average hourly wage in U.S. dollars that was observed in a given study and was consequently adjusted for the year 2021 using the inflation rate. Observations in currencies other than U.S. dollars were converted with the exchange rate at the time the data in the study were collected. If the observation was not assigned to a specific region or country, the international inflation rate was used (Source for inflation rates: International Monetary Fund).
Data points	Number of data points on which the hourly wages in a study are based. A data point can be the answer to a survey question or the calculated hourly wage of a specific task, that was obtained through technical data collection methods.
Wage std. dev	Standard deviation of Hourly Wage and Hourly Wage 2021 that is reported in a given study or was obtained from the authors of the study.

To calculate and weight the mean hourly wages for a specific category of crowdwork and to make the respective statistical adjustments (for more details, see Sect. 3.3.), we obtained the number of data points (*Data Points*) and the standard deviation (*Wage Std. Dev.*) for *Hourly Wage 2021*. For 11 hourly wages, we needed to calculate the number of data points per observation, for example, from a confidence interval or by percentages. Overall, we were able to obtain or calculate the standard deviation for 85 hourly wages.

To enable a more nuanced analysis of wages for different forms of crowdworking, we created the categorical variable *Crowdworking Category*, which equals 1 if the observation results from microtasks, 2 if it results from online freelancing, and 3 if it results from a crowd contest. The coding of the data points is based on the framework described in Sect. 2. In particular, we checked whether a platform was previously assigned to one of the three categories in the literature (e.g., Berg et al. 2018; Schmidt 2016) and whether the platform itself states that it is active in one of the three crowdworking categories. To show the effect of unpaid work, we create the dummy variable *Involves Unpaid Work*, which equals 1 if the reported hourly wage in a study considers unpaid work and 0 otherwise.

To examine how wages are determined in the three crowdworking categories and the extent to which the data collection method influences the wages estimated, we define the dummy variable *Data Collection Method*. This variable equals 1 if the hourly wage was estimated through a technical data collection method and 0 if the authors used a survey.

### Method

To analyze hourly wages in the different crowdworking categories, we define five groups of hourly wages present in empirical studies: the hourly wages of microtask workers, the hourly wages of microtask workers considering unpaid work, the hourly wages of online freelancers, the hourly wages of online freelancers considering unpaid work, and the hourly wage of workers participating in crowd contests considering unpaid work.^8^ Thus, we calculate average hourly wages for different crowdwork categories, while considering the effect of studies that account only for paid work and those that also account for unpaid work. We then calculate the mean and standard deviation for each group to compare the resulting mean hourly wages, using a two-sample t-test for unequal variances and Satterthwaite’s (1946) formula as an approximation for the needed degrees of freedom. As sample sizes, we use the respective number of hourly wages per group.

We use the variable *Crowdworking Category* and the outlined method to examine the differences among wages in microtasks, online freelancing, and crowd contests*.* We further assess the effect of hourly wages earned by workers in the categories microtasks and online freelancing conditional on whether the studies account only for paid work or also consider unpaid work, using the dummy variable *Involves Unpaid Work*. Finally, we examine the potential effect of the data collection method on the estimated hourly wage using the dummy variable *Data Collection Method*.

To account for the sophistication of the respective studies and the precision with which the hourly wages are measured, we treat *Hourly Wage 2021* as the quasi-effect size in our meta-analysis and calculate weighted means of hourly wages. First, we weight hourly wages by the number of data points (*Data Points*) in the respective study to account for the sophistication of the particular study. Second, we weight our observations by the inverted variance of an average hourly wage that was reported in the respective study ($\frac{1}{{Wage Std. Dev.^{2} }}$) to account for various degrees of precision of the hourly wages.^9^ These two weights are commonly used in meta-analyses (Schmidt and Hunter 2015; Lee et al. 2016) and allow us to give greater weight to observations based on many data points and observations with a small variance, which presumably provides more consistent estimates of the true hourly wage in the crowdworking population. In what follows, we use the abbreviations *n-weighted-mean* for hourly wages weighted by the number of observations and *v-weighted-mean* for hourly wages weighted by the inverted variance, to distinguish the two ways of weighting hourly wages in our sample. In line with prior meta-analyses in the field of crowdworking (Spindeldreher and Schlagwein 2016), we calculate the mean of *Hourly Wage 2021* only if observations from at least five independent studies are available.

## Results

### Descriptive Statistics

We report the summary statistics for the full dataset and independently for the subsamples of microtasks, online freelancing, and crowd contests. Many observations come from the crowdworking categories microtasks (n = 55) and online freelancing (n = 47), while only three come from the crowd contest category. Table 4 provides summary statistics for the variables of interest. We find the observations in our dataset to be balanced in terms of the data collection method, with roughly half the hourly wages in the full dataset coming from surveys and the other half from technical methods. However, hourly wages of online freelancers are mainly measured through technical methods, while workers performing microtasks are often evaluated through surveys. While approximately one-third of all observations account for unpaid work, only one-fifth of the hourly wages of online freelancers account for unpaid work.

**Table 4:** Summary statistics

Full dataset						Subsample microtasks (n = 55)		Subsample online freelancing (n = 47)		Subsample crowd contest (n = 3)	
Variable	Mean	SD	Min	Median	Max	Mean	SD	Mean	SD	Mean	SD
Data Collection Method	0.46	0.51	0	0	1	0.22	0.42	0.77	0.43	0	0
Category	1.50	0.56	1	1	3	–	–	–	–	–	–
Involves Unpaid Work	0.37	0.49	0	0	1	0.51	0.50	0.17	0.38	1	0
Hourly Wage 2021	13.92	16.01	1.48	5.97	85.11	4.43	2.46	25.17	18.31	11.80	2.47
Data Points	731.10	1746.37	14	252	12,326	897.02	2195.43	579.60	1073.03	62.67	67.45
Wage Std. Dev. ^a^	12.57	13.52	0.71	5.90	71.04	5.85	6.99	19.97	15.29	8.65	2.19

^a^For the full dataset, we could measure standard deviations for only 86 of the 105 observations. For the subsample microtasks, we could measure the standard deviation for 44 of the 55 observations. For the subsample online freelancing, we could measure the standard deviation for 41 of the 47 observations. For the subsample crowd contests, we could measure the standard deviation for 2 of the 3 observations

**Fig. 5:**
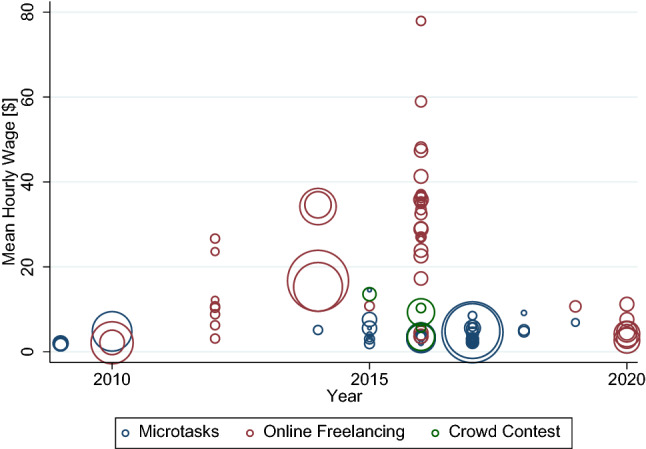
Scatterplot of wages from 22 primary studies. The size of each circle represents the number of observations relative to the other observations

We show the distribution of the hourly wages per year for microtasks, online freelancing, and crowd contests in Fig. 5. Circles indicate one observation – namely, an average hourly wage reported in the respective study. The larger the circles, the higher the number of data points on which the wage is based. Notably, most observations fall in the year 2016. By running a simple regression of hourly wages on the year, we observe a negative but statistically non-significant correlation. Thus, hourly wages remain stable and even decline over time, which might reflect an increase in competition among workers and platforms. We again document the broad range of reported hourly wages in Fig. 5 to highlight the necessity of assessing the various categories of crowdworking.

### Mean Hourly Wages

Although a multiple regression might be a natural method to determine the factors influencing hourly wages, we do not suggest that our empirical analysis reflects causal relationships. We therefore decided to calculate and statistically test the differences between mean hourly wages of the different groups. Table 5 reports the mean hourly wages for the five groups of hourly wages present in empirical studies. We present the results respectively by adjusting wages by the number of data points in a study and by accounting for unpaid work. In the case of a v-weighted hourly wage for online freelancing considering unpaid work, we could only find two independent studies, while for workers participating in crowd contests, we had fewer than five observations overall. In line with our inclusion criteria, we therefore did not calculate either a v-weighted mean hourly wage for freelancers accounting for unpaid work or a mean hourly wage for workers participating in crowd contests.

**Table 5:** Mean hourly wages

Category	Not considering unpaid work	Considering unpaid work
(a) n-weighted, adjusted 2021		
Online freelancing	***$20.88*** (SD: $14.31) (Data points: 23,931) (Observations: 39)	***$4.87*** (SD: $2.60) (Data points: 3310) (Observations: 8)
Microtasks	***$5.55*** (SD: $1.05) (Data points: 34,045) (Observations: 15)	***$4.07*** (SD: $1.55) (Data points: 8452) (Observations: 18)
Crowd contest	–^a^	–^a^
(b) v-weighted, adjusted 2021		
Online freelancing	***$12.13*** (SD: $7.87) (Data points: 20,164) (Observations: 37)	–^a^
Microtasks	***$4.97*** (SD: $2.04) (Data points: 29,447) (Observations: 21)	***$3.78*** (SD: $3.35) (Data points: 6550) (Observations: 13)
Crowd contest	–^a^	–^a^

*Data points* is the number of data points used to calculate the respective mean hourly wage. Each data point represents either the response to a survey question about the wage of a crowdworker or a wage collected via a technical data collection method on the respective platform

*Observations* is the number of observations used to calculate the respective mean hourly wage. Each observation is the average hourly wages reported in a given study for a sample of crowdworkers

^a^Less than 5 independent observations

**Fig. 6:**
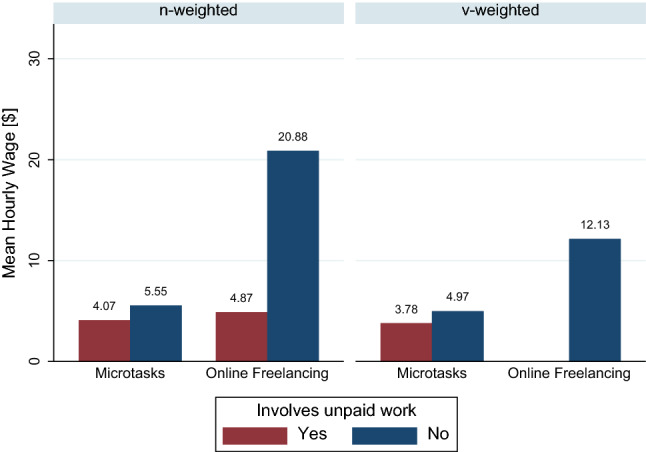
Comparison of calculated mean hourly wages

An overview of the estimated hourly wages for microtasks and online freelancers appears in Fig. 6. We find that hourly wages of workers completing microtasks range from $3.78 to $5.55 per hour, depending on the weighting method and whether unpaid work is taken into account. With up to $20.88 per hour, we find that the calculated hourly wage of online freelancers in our meta-analysis significantly exceeds that of workers doing microtasks when unpaid work is neglected, as Panels A and B (line 1) of Table 6 show. We also find that the v-weighted means are always lower than their n-weighted counterparts. The difference between the n- and v-weighted mean is especially high for online freelancers, which can be partly attributed to the observations stemming from the studies of Barzilay and Ben-David (2017) and Dunn (2017). Both studies report relatively high wages, but also high standard deviations, even though their hourly wage estimations are based on thousands of data points. As a result, the hourly wages from these studies are weighted more heavily for the n-weighted mean than for the v-weighted mean. This example highlights the importance of using both the number of underlying data points and the inverted variance as weights to estimate the true hourly wage in crowdworking.

**Table 6:** Comparison of means

Comparison	Mean	Difference	No. of data points	No. of observations
Panel A: Comparison of n-weighted means, adjusted 2021				
(1) Online freelancing paid/microtasks paid	$20.88	− $15.33***	23,931/34045	39/15
(2) Online freelancing unpaid/microtasks unpaid	$4.87	− $0.80	3310/8452	8/18
(3) Online freelancing paid/online freelancing unpaid	$20.88	− $16.01***	23,931/3310	39/8
(4) Microtask paid/microtasks unpaid	$5.55	− $1.48***	1050/1550	15/18
(5) Technical/survey	$12.54	− $7.60***	52,476/15144	42/41
Panel B: Comparison of v-weighted means, adjusted 2021				
(1) Online freelancing paid/microtasks paid	$12.13	− $7.16***	20,164/29447	37/21
(2) Online freelancing unpaid/microtasks unpaid	^a^		1988/6550	3/13
(3) Online freelancing paid/online Freelancing unpaid	^a^		20,164/1988	37/3
(4) Microtasks paid/microtasks unpaid	$4.97	− $1.19	29,447/6550	21/13
(5) Technical/survey	$5.51	− $1.70**	43,562/11280	39/30

*p < 0.10%, **p < 0.05, ***p < 0.01

^a^Less than five independent observations

Furthermore, we find that for microtasks, almost three out of four datapoints are collected via plugins, while only one in four datapoints for online freelancers is extracted through such technical data collection methods. We find that wages estimated by technical data collection are higher than wages collected through surveys, as the comparison of the mean hourly wages in Panels A and B (line 5) of Table 6 shows. Studies such as that of Bayudan-Dacuycuy and Kryz Baje (2021), which had direct access to primary platform data, show that even more complex remuneration and work processes can be measured through technical methods, especially when companies grant researchers access to their platform. However, it should be noted that these often better-paid online freelancing jobs also entail more unpaid work, which is often neglected by this type of data collection.

### Factors Determining the Hourly Wage of a Crowdworker

In the reviewed studies, we find four factors that might determine the heterogeneity of the observed wages. These factors are the skills of a specific worker, sample differences in crowdworker demographics, the current state of the crowdworking market, and whether or not a study accounts for unpaid work.

First, the **skills of a crowdworker** consist of at least two elements: the acquired skills that set the worker apart from others (Bayudan-Dacuycuy and Kryz Baje 2021; Braesemann et al. 2021) and the experience gained by spending time on crowdworking platforms. While labor economics suggest that better-skilled workers earn higher wages, research has also shown that crowdworkers become more efficient over time, enabling them to earn higher wages (Rani and Furrer 2019; Sannon and Cosley 2019). For example, in the crowdworking category of microtasks, workers use plugins to become more efficient, or they search in forums to find better-paying jobs (Kingsley et al. 2015; Silberman and Irani 2015). In online freelancing, workers often build a reputation, which also allows them to earn higher wages (Aleksynska et al. 2019; Haidar and Keune 2021).

The second factor determining hourly wages is **sample differences in crowdworker demographics**. Age and gender in particular play a major role in the remuneration of crowdworkers. Younger workers earn more than their older colleagues, and men earn more than women (Litman et al. 2020; Caro et al. 2021). With regard to age, younger workers are less likely to be married or have children, which may give them more flexibility in working hours (Litman et al. 2020; Wood et al. 2019a). In general, women are less experienced in crowdworking and also more involved in care work (Litman et al. 2020). As a result, they must often take on less favorable tasks at off-peak times, because most tasks are posted during the working hours of large requesters, such as those in the United States (Berg et al. 2018; Gerber 2022). The location of crowdworkers is also important because high-paying tasks are often restricted to workers in a specific country, which is mostly in the developed world (Lehdonvirta et al. 2019; Rani and Furrer 2021). In 86% of cases in which a specific country is requested, international requesters ask for U.S. workers (Difallah et al. 2015). Health problems can also affect the pace of work completion and, thus, the hourly wage (Caro et al. 2021).

The third factor affecting hourly wages is the current **supply and demand for tasks and workers** (Nikzad 2017; Bayudan-Dacuycuy and Kryz Baje 2021; Zhang et al. 2022). Muszyński et al. (2021) contend that the smoothing of labor supply and demand through employer–employee relationships is absent in crowdworking. Workers completing microtasks, in particular, are vulnerable to low hourly wages caused by labor oversupply, as microtasks require few skills and therefore have a low barrier to entry (Braesemann et al. 2021). For many crowdworkers, however, microtasks could represent a gap-filling activity that is easily carried out between other activities and which therefore also pays a comparatively low wage (Teevan 2016; Newlands and Lutz 2021). During the COVID-19 pandemic, increasingly more people turned to crowdwork as a source of income, thereby lowering average wages through increased competition and supply (Stephany et al. 2020; Braesemann et al. 2021; Muszyński et al. 2021). However, even before the pandemic, Graham and Anwar (2019) identified a large oversupply of labor on one of the largest platforms for online freelancers worldwide (Kässi and Lehdonvirta 2018). Although demand for online crowdworkers has now exceeded pre-pandemic levels (Stephany et al. 2020), an oversupply of workers for most tasks is still likely.

Finally, the hourly wage also depends on **how much unpaid work is done** and whether the empirical **studies account for unpaid work**. Unpaid work involves communicating with requesters, searching for tasks, building a reputation, writing reviews, and beginning tasks that the worker will not complete (Berg et al. 2018; Pulignano and Marà 2021; Rani and Furrer 2021; Toxtli et al. 2021; Lascău et al. 2022). Rejected work also increases the extent of unpaid work. Depending on the platform, up to 15% of all work is rejected (Berg et al. 2018). The fact that on most platforms requesters are allowed to keep the results of rejected work while the worker receives no remuneration for the task at hand is a clear indication of the prevailing power asymmetry between workers and requesters (Beerepoot and Lambregts 2015; Berg et al. 2018; Lascău et al. 2022). Research also suggests that non-native English speakers take more time to complete a task (Toxtli et al. 2021) and have a higher rejection rate, as misunderstandings more often lead to low-quality results (Goodman et al. 2013; Chandler and Shapiro 2016).

The estimated amount of unpaid work in crowdworking varies depending on the category of crowdwork. Rani et al. (2021) report that 38% of all work done on freelancing platforms and 33% of work done in microtasking is uncompensated. In another study, Rani and Furrer (2021) estimate that 23% of all work is unpaid, while Wood et al. (2019a) report that 39% of the work done by crowdworkers is unpaid. We confirm these estimates at least for microtasks, with between 23 and 26% (Table 6, Panels A and B) of all work being unpaid in our aggregated data.

## Discussion

Our main results show estimated hourly wages of less than $6 for workers completing microtasks when unpaid labor is not accounted for and approximately $4 when this is the case. For the more diverse domain of online freelancing, we find hourly wages as high as $20.88 per hour, but our estimates fall off sharply when we account for unpaid work, to an average hourly wage of $4.87. These results are consistent with previous research, which estimates that the proportion of unpaid work is higher for online freelancers than for microtasks (Pulignano and Marà 2021; Wood et al. 2019a). We also show how the different complexity of remuneration and work processes can dictate the choice of data collection method in crowdworking research. In this context, we again emphasize that unpaid work is usually neglected when technical data collection methods are used and call for future research to improve these methods in that respect.

We also note that skills are not the only relevant factor in determining the hourly wage a crowdworker can earn. Using microtasks as gap fillers (Bayudan-Dacuycuy and Kryz Baje 2021) helps answer the question of why some crowdworkers are not systematically moving into the higher-paying domain of online freelancing by learning required skills (Stephany 2021). Nonetheless, some crowdworkers do indeed use low-entry tasks to build skills necessary to transition to online freelancing (Kuek et al. 2015). Importantly, much of the work done is completed by a small proportion of skilled microtask workers (Chandler and Shapiro 2016; Codagnone et al. 2016) who can earn incomes well in excess of local minimum wages (Heeks 2017; Berg and Rani 2021). For example, constantly checking profiles of known requesters for new assignments allows these well-trained microtask workers to earn wages closer to an average of $11 per hour (Hara et al. 2018).

### Contribution to Current Policy Debates

Policy debate over the hourly wages paid in crowdworking is ongoing (Berg 2016; Leimeister et al. 2016; O’Higgins and Caro 2022). We consider three discourses in this debate originally identified by Greef et al. (2017). First, scholars have engaged in discourse about the general *transformation of work*, from location-based work to online web-based platforms (Rani et al. 2021). Second, there is discourse on *growth and competition*, which evaluates the future potential of online labor markets, but also the already-fierce competition between workers for wages and completion times on crowdworking platforms (Pongratz and Bormann 2017). The third discourse centers on the issue of *social security and the participation of workers* in shaping the future of their work environment. Here, discourse involves how and in what form agencies such as trade unions can condemn but also change poor working conditions, such as the lack of social security (Johnston and Land-Kazlauskas 2019).

Our study contributes to the general discourse about the transformation of work, especially in the field of the ever-increasing information asymmetry in crowdworking (Agrawal et al. 2015; Aloisi 2015). While online labor platforms monitor workers in ways often unthinkable in traditional work environments (Wood et al. 2019b), the workers themselves are left with third-party browser plugins, to track their remuneration and performance. In most cases, legislators and trade unions do not have access to these kinds of technically obtained data and therefore must use surveys to monitor hourly wages and other important key figures of work (Serfling 2018). For more established industries, researchers can often also rely on official statistics collected by government agencies, which is not yet the case for crowdworkers. Some scholars therefore suggest granting legislators access to anonymized transaction data from online labor platforms, to help policy makers regulate the crowdworking market (Heeks 2017; European Commission 2021). We contribute to this debate by increasing the transparency in mean hourly wages, based on multiple studies and data collection methods. Regarding the data collection method, researchers should be aware of how different methods can affect their results. We find large differences between hourly wages estimated through surveys and technical data collection methods. Future research should consider the effect of unpaid work on estimated hourly wages, for example, in the form of a correction subtracted from the estimated hourly wage that only accounts for paid work. Projects such as Fair Crowd Work and Fairwork could quantify the proportion of unpaid work and calculate and publish a correction factor accordingly for each platform. As we show in our study, the effects of unpaid work are significant and should not be neglected.

With the mean hourly wages reported in our study, we also contribute to the discussion on the organizational transformation of the relationship between online labor market platforms and crowdworkers (Dengler and Matthes 2015; Drahokoupil and Fabo 2016). Because of the low wages paid to workers completing microtasks, research often argues that these workers are overdue for legal classification as salaried employees (Berg 2016), as the dependency between workers and platforms is partly comparable to dependent employees (Preis 2016; Leist et al. 2017). Under current laws, however, the classification of crowdworkers into the common categories of labor law is difficult and controversial (see, e.g., Otey v. CrowdFlower, Inc. 2013). In addition to these problems, regulating and monitoring the crowdworking market is difficult for legislators because of the transnational nature of the markets, the heterogeneity in platforms, and the differing dependencies between workers and platforms (Greef et al. 2017; Serfling 2018). In our study, we deal with this heterogeneity by analyzing the specific area of online crowdworking, which allows us to make meaningful distinctions among three categories of crowdwork. In these categories, we find significant wage differences, which again highlights the importance of a precise definition of the investigated categories of crowdworking in future research and the policy debate. Especially in the field of crowd contests, data on wages are sparse and thus offer an important avenue for future research. Empirical studies would also benefit from using data collection methods other than surveys, which have so far been the only method to understand the complex remuneration and work process in crowd contests.

We also contribute to the second discourse focusing on the growth and development of online labor markets, which is strongly connected with the call for additional research in the field of crowdworking (Greef et al. 2017; Maier and Viete 2017). With the novel dataset used in this study, we aggregate information on the main motivator of crowdworkers (Kaufmann et al. 2011; Goodman et al. 2013; Lioznova et al. 2020) and contribute to existing efforts to extend the database on online crowdworking. Furthermore, the results of our analysis could serve as a preliminary benchmark for future studies examining the wages of new online workers in the wake of global pandemics and migration movements.

Our findings are especially relevant to the debate on the social security and participation of crowdworkers in decision-making processes regarding the platforms on which they work (Preis 2016). In most countries and on the majority of platforms, neither social security nor options for participation exist. Many researchers have therefore criticized the working conditions in crowdworking, with some even describing them as precarious (Kittur et al. 2013; Schriner and Oerther 2014; Hara et al. 2018; Whiting et al. 2019). As workers are mostly not employed by the platforms, but labeled “contractors” or “freelancers,” online labor market platforms are not responsible for paid leave, maximum working hours, or mandatory breaks (Barzilay and Ben-David 2017).

Because of unclear governance mechanisms and information asymmetries, platforms are particularly prone to contribute to precarious work conditions (Cutolo and Kenney 2019; Khovanskaya et al. 2019; Gegenhuber et al. 2021). In this case, trade unions can act not only as strong negotiators on behalf of the workers, as in traditional labor markets, but also as an institution that could facilitate the necessary communication and exchange between workers (Johnston and Land-Kazlauskas 2019). With our meta-analysis, we substantiate the criticism of low wages, at least in the area of microtasks, for which we consistently find mean hourly wages of under $6 per hour. Given this mean wage and the lack of health insurance for the majority of workers, it is clear why many workers have called for better or even any social benefits (Wood et al. 2019b). In online freelancing, however, we calculate mean hourly wages, which are much higher than those for workers completing microtasks, with calculated mean wages up to $20.88 per hour. However, when considering unpaid work, the wages of online freelancing are relatively low.

It is also important to note that these wages should not be considered only from a Western and industrialized country perspective (Casilli 2016; Elbanna and Idowu 2021). Given the prices of particular goods at different locations, it is understandable why an hourly wage of $1–$2 is more attractive to a Kenyan than a U.S. citizen (De Groen and Maselli 2016; Bayudan-Dacuycuy and Kryz Baje 2021; Berg and Rani 2021). Considering the concept of purchasing power parity, Beerepoot and Lambregts (2015) determined higher relative wages for online workers from India and the Philippines than U.S. workers. However, with around two-thirds of all observations in our meta-analysis being from U.S. workers, crowdworking research seems to suffer from white, educated, industrialized, rich, and democratic (WEIRD) sample bias (Henrich et al. 2010).^10^ Therefore, to counter criticism of the limited external validity, future research could also more extensively examine crowdworkers from the Global South and migrant crowdworkers.

In recent years, platforms’ governance has also improved, such as through the introduction of a minimum hourly wage on oDesk (now Upwork) (Heeks 2017). Whereas Hanrahan et al. (2021) observed an increase in wages from 2018 to 2019, we find a small, albeit statistically non-significant, decrease in hourly wages in our dataset. The difference might be due to the inclusion of many observations that take unpaid work into account, while Hanrahan et al.’s results are based on observations only considering paid work. In principle, the advantages of crowdworking should not be neglected. For example, the high degree of spatial and temporal flexibility when completing work as a crowdworker is one of the greatest advantages of crowdwork (Brandt et al. 2016; D'Cruz and Noronha 2016; Berg et al. 2018). Thus, crowdworking could potentially facilitate the participation of people with disabilities or individuals with care obligations in the labor market (Adams and Berg 2017; Hara et al. 2019).

### Limitations

Any meta-analysis can suffer from publication bias; that is, some studies are not published because of the non-significance or direction of their results and therefore cannot be considered. For our meta-analysis, common methods for discovering publication bias, such as funnel plots (Elvik 1998; Ahmed et al. 2012), are not adequate empirical techniques because we do not calculate effect sizes such as Hedges’s *g* or Cohen’s *d* but rather calculate average hourly wages as a quasi-effect size (Song et al. 2000).

Not using the standard effect sizes and simply considering the reported hourly wages has two implications. First, we do not expect a symmetric distribution of hourly wages, which we would expect when measuring effect sizes and reporting a funnel plot. Previous research has shown that the distribution of wages on crowdworking platforms is right skewed (Berg 2016; Adams and Berg 2017; Kaplan et al. 2018). In other words, the majority of workers completing microtasks and online freelancers earn only small wages, while few earn wages that are many times higher than the average hourly crowdworking wage. We found that the distribution of hourly wages in our data sample was right skewed as well, which indicates that we observe a representative distribution. Second, whether the reason for publication bias regarding effect sizes can be transferred to the estimation of hourly wages is questionable. Publication bias frequently occurs when only statistically significant results are published, while research resulting in statistically non-significant results remains unpublished (Bozarth and Roberts 1972). In our case, the risk of not reporting non-significant results should be minimal, because most studies in the domain of crowdworking do not conduct tests for statistical differences in wages.

Another problem when conducting a meta-analysis is the potential lack of internal validity (Brutus et al. 2013); that is, a single study should not dominate hourly wage estimates. To be able to average out study-specific effects, such as the particular sample or the data collection method used, we only calculated an hourly wage if at least five independent studies were available in our meta-analysis (Spindeldreher and Schlagwein 2016).

Furthermore, research on crowdworking wages faces a high degree of heterogeneity, especially when considering online freelancing, as the high standard deviation for the mean hourly wage evidences. Our mean hourly wages are therefore only valid for a specific category of crowdwork and not crowdwork in general. We tried to counteract this problem by thoroughly separating crowdwork into three prominent categories. For microtasks, which tend to be homogeneous, standard deviations in our sample are substantially smaller, which indicates a more precise estimate of mean hourly wages. To make wages more comparable over time, we adjusted them for inflation, but we recognize that wage bargaining and wage increases may not always follow changes in consumer prices (Blanchflower et al. 2017; Lübker 2020), especially in the domain of crowdworking. If so, we would have overestimated the inflation-adjusted wages in our meta-analyses at best, because wages in crowdworking have not risen as fast as inflation rates. If workers or unions perceive wages as unethical and too low, they might in fact be even lower if wages have not increased with the inflation rate.^11^ Nevertheless, the inflation rates in Europe and the United States were low during the observation period of our meta-analysis (Forbes et al. 2021; Koester et al. 2021). Finally, meta-analyses include older studies by nature, even though the results obtained in a particular study may not be fully comparable with those from more recent periods.

## Conclusion

This meta-analysis investigates 105 mean hourly wages in crowdwork that were reported in 22 different studies. We extend the literature by estimating the mean hourly wages for different categories of crowdworking, while also considering the method of data collection and the effect of unpaid work. Our investigation of mean hourly wages is not limited to a single platform, region, or data collection method, which further raises transparency for workers, researchers, and legislators (De Groen et al. 2016; Litman et al. 2020; Wong et al. 2020). Our results, to our knowledge, are based on the most comprehensive dataset on hourly wages in crowdworking in recent literature. We show that working on microtasks results in wages ranging from $3.78 to $5.55 per hour on average. Online freelancers earn $4.87 to $20.88 per hour on average, which is up to three times more than microtask workers.

Future experimental research should test the influence of data collection methods that might result in self-reporting bias in crowdworking wages. For example, researchers could monitor crowdworkers’ hourly wages through a plugin and then ask them about their earnings. Quantifying the potential difference between the different methods of data collection is especially important when evaluating the wages of online freelancers, as the unpaid portion of the work is likely to be higher and could be underestimated. Policy makers should be aware that the use of surveys instead of technical data collection methods could change the estimation of wages. In recent literature, the method of data collection strongly depends on the willingness of the specific crowdworking platform to share its data with researchers (Agrawal et al. 2015; Bertschek et al. 2016; Barzilay and Ben-David 2017). If an online freelancing platform decides not to share its data or only shares out-of-date data, surveys become the only option to investigate the current wages of workers. Surveys could also turn out to be the only viable option to examine wages on crowd contest platforms, a field in which estimates of hourly wages are sparse (De Groen and Maselli 2016).

As we found a significant difference in wages accounting and not accounting for unpaid work, we suggest that researchers investigating hourly wages in crowdworking in the future always report an hourly wage that also accounts for unpaid work. Because most researchers assume that one-third to one-half of the work time is unpaid (Berg et al. 2018; Rani et al. 2021), which is in line with our meta-analysis results, a wage correction factor that does take unpaid work into account should be around 20–33%. Such a wage correction factor for hourly wages that considers unpaid components would make hourly wages from different crowdworking studies more comparable. Furthermore, we encourage researchers to assess crowdworkers from countries other than the United States. Undertaking a broader comparison of wages between different regions and considering the prices of goods and services at different locations might provide a more refined picture of crowdworking as a new online labor market.

## Supplementary Information

Below is the link to the electronic supplementary material.

Supplementary file1 (PDF 12 kb)

## Footnotes

1 Other categories of crowdworking include *crowd complementor* and *collaborative community* platforms (e.g., Boudreau and Lakhani 2013). To make the analysis concise, because jobs in collaborative communities such as Innosabi or Wikipedia are often unpaid (Hornuf and Jeworrek 2022) and because of the dearth of empirical studies on wages for other categories such as crowd complementors, we limit our analysis to microtasks, online freelancing, and crowd contests.

2 Keeping track of their productivity can affect the effectiveness of crowdworkers' work, which may lead to a systematic bias of the wages collected through plugins.

3 Appendix A reports the full list of keyword combination.

4 We cannot assume that the number of data points are identical to the number of individual crowdworkers for three reasons. First, crowdworkers often work on multiple platforms (Serfling 2018), which allows them to answer surveys on different platforms and potentially results in two separate data points for one crowdworker. Second, even crowdworkers who only perform tasks on one platform could potentially answer several of the included surveys, as the surveys were conducted by different researchers and at different points in time, which could again result in multiple data points per worker. Arguably, given the large number of crowdworkers on a platform such as MTurk, it seems rather unlikely that one crowdworker would undertake the same task of filling out a survey twice. Third, technical data collection methods often estimate the hourly wage by determining the wage per task and not per worker. Because workers often complete multiple tasks on one platform, the number of observed tasks is not equal to the number of observed workers.

5 China, Germany, India, Italy, Philippines, Serbia, Ukraine, and United States.

6 Advego.ru, MTurk, Clickworker, CoContest, Crowd Guru, CrowdFlower, EPWK, fl.ru, Freelance.ru, Freelance.ua, Free-lance.ua, Freelancehunt.com, Freelancer.com, Kabanchik.ua, k68, Microworkers, Prolific, Upwork.com, Weblancer.net, ZBJ, 680, and 99designs.

7 We obtained hourly wages for workers from 2009 to 2020; however, we did not find any studies that collected data in 2011 and 2013.

8 No study considered only paid work for crowd contests.

9 Most studies report the standard deviation of the hourly wage, which we then converted to the variance.

10 By contrast, Rani and Furrer (2021) investigate microtask workers from developing countries; Wood et al. (2019a) also examine workers from Sub-Saharan Africa, and Beerepoot and Lambregts (2015) assess workers from the Philippines.

11 Furthermore, arguments that workers in surveys erroneously overestimate their hourly wages to protect their self-image (Mazar et al. 2008) are hardly valid, as research has shown that crowdworkers are well aware of their often low hourly wages (D'Cruz and Noronha 2016; Whiting et al. 2019).
