# Supplementary material for: Hourly Wages in Crowdworking: A Meta-Analysis
Source: Bus Inf Syst Eng. 2022 Aug 30;64(5):553–73. doi: 10.1007/s12599-022-00769-5 (PMC9425816; doi:10.1007/s12599-022-00769-5)
Supplement: Supplementary file 1 — Supplementary file1 (PDF 12 kb) [file 12599_2022_769_MOESM1_ESM.pdf]

Hourly Wages in Crowdworking: A Meta-Analysis

Lars Hornuf, Daniel Vrankar

Business & Information Systems Engineering (2022)

Appendix (available online via http://link.springer.com)

Appendix A: List of Keyword Combination

Table A1 Keyword combinations

Base word	Addendum
"crowdwork<br>"crowdsource	per hour"
	hourly"
	wage"
	remuneration"
	earned"
	earnings"
	pay"
	$"
	dollar"
	USD"
	€"
	EUR"
	¥"
	yen"
	£"
	pound"
"Crowdwork<br>"Crowdsource	Entlohnung"
	Mindestlohn"
	Stundenlohn"
	Einkommen"
	Vergütung"
	Pfund"

The base word is combined with the addendum. For example, the first keyword combination we searched for was "crowdwork per hour".
